# Reducing uncertainty in health-care resource allocation

**DOI:** 10.1038/sj.bjc.6603795

**Published:** 2007-05-22

**Authors:** T Simonsson, K Sjölund, P Bümming, H Ahlman, B Nilsson, A Odén

**Affiliations:** 1 Department of Biomedicine, Sahlgrenska Academy at Göteborg University, Göteborg SE-405 30, Sweden; 2 Department of Surgery, Lundberg Laboratory for Cancer Research, Sahlgrenska Academy at Göteborg University, Göteborg SE-413 45, Sweden; 3 Department of Mathematical Sciences, Chalmers University of Technology, Göteborg SE-412 96, Sweden

**Keywords:** survival, treatment, quality of life, imatinib mesylate, gastrointestinal stromal tumour, GIST

## Abstract

A key task for health policymakers is to optimise the outcome of health care interventions. The pricing of a new generation of cancer drugs, in combination with limited health care resources, has highlighted the need for improved methodology to estimate outcomes of different treatment options. Here we introduce new general methodology, which for the first time employs continuous hazard functions for analysis of survival data. Access to continuous hazard functions allows more precise estimations of survival outcomes for different treatment options. We illustrate the methodology by calculating outcomes for adjuvant treatment of gastrointestinal stromal tumours with imatinib mesylate, which selectively inhibits the activity of a cancer-causing enzyme and is a hallmark representative for the new generation of cancer drugs. The calculations reveal that optimal drug pricing can generate all win situations that improve drug availability to patients, make the most of public expenditure on drugs and increase pharmaceutical company gross profits. The use of continuous hazard functions for analysis of survival data may reduce uncertainty in health care resource allocation, and the methodology can be used for drug price negotiations and to investigate health care intervention thresholds. Health policy makers, pharmaceutical industry, reimbursement authorities and insurance companies, as well as clinicians and patient organisations, should find the methodology useful.

Cancer includes a wide spectrum of diseases that place a massive economic burden on society. It is currently estimated that one in three will be diagnosed with cancer at some time in life, that one in four will die from cancer and, although cancer diseases are more common among the elderly, their relative contribution to disability and death in younger people remains high (Boyle and Ferlay, 2005; Danaei *et al*, 2005; Ries *et al*, 2005).

All forms of cancer have two features in common; alterations to the genetic code and unrestricted cell growth, the latter being a consequence of the former. Since the latter also constitutes the point of attack for the vast majority of current cancer therapies, normal cells are essentially affected in the same way as cancer cells. This makes precise dosage very difficult and calls for development of more specific therapies.

The recent completion of the human genome project (Lander *et al*, 2001) has prompted a paradigm shift towards medicine characterised less by treating symptoms and more by looking to the fundamental causes of disease. Detailed knowledge about the molecular origins of disease now allows rational drug design. A new generation of cancer drugs, which selectively inhibits the activity of cancer-causing genes or their protein products, is just reaching the market, but recent reports question the pricing and availability of new cancer drugs (Berenson, 2005; Goldman, 2005; Smith, 2005; Wilking and Jonsson, 2005) and have highlighted a need for improved methodology to estimate benefits of health care interventions.

A general method to estimate benefits of health care interventions is to predict the expected gain in quality adjusted life years (QALYs) (Drummond *et al*, 1997), which take into account both quantity and quality of life generated by health care interventions. QALYs provide a common currency to assess the extent of the benefits gained from a variety of interventions in terms of health related quality of life and survival for the patient. When combined with the cost for providing the interventions, cost–utility ratios result. These indicate the additional cost to generate a year of perfect health (one QALY). Comparisons can be made between interventions, and priorities can be established based on those interventions that are relatively inexpensive (low cost per QALY) and those that are relatively expensive (high cost per QALY). The use of QALYs in resource allocation decisions thus means that choices between different patient groups competing for medical care are made explicit and health care policymakers are given an insight into the likely benefits from investing in new technologies and therapies. A key question is whether the expected gain in QALYs outweighs the costs associated with the development of more specific treatments. QALYs are nonetheless far from perfect as a measure of outcomes, with a number of technical and methodological shortcomings. Improvements in disease management hence call for better methods to predict the expected gain in QALYs.

Survival data are commonly analysed by Cox regression (or proportional hazards regression), and results are often presented as simple hazard ratios. Distinct limitations of this methodology are that the resulting hazard functions are discontinuous, that the influence of time will not be explicitly modelled and that the hazard ratios are assumed to be constant over time. However, multidimensional hazard functions can be determined in a way that they are continuous in all time variables. Here we introduce the use of continuous hazard functions for QALY calculations. We show that special Poisson regression models allow hazard functions to be estimated as continuous functions, which makes it possible to derive and calculate probabilities, expected length of life and expected gain in QALYs.

An urgent question for health policymakers is whether the new generation of cancer drugs will provide value for money in comparison to other health care interventions. Gastrointestinal stromal tumours (GISTs) are non-epithelial tumours of the gastrointestinal tract that are commonly caused by activating or gain-of-function mutations in the *KIT* gene (Hirota *et al*, 1998). If surgery failed, GISTs were inevitably fatal until the discovery of the tyrosine kinase inhibitor imatinib mesylate (Gleevec®) (Buchdunger *et al*, 1996; Joensuu *et al*, 2001; Tuveson *et al*, 2001). Imatinib mesylate reduces GIST cell proliferation by binding to the catalytic pocket of the *KIT* protein product (Schindler *et al*, 2000), and serves as a hallmark representative for the emerging generation of cancer drugs that selectively inhibits the activity of cancer causing genes or their protein products. We illustrate the new methodology by calculating the expected gain in QALYs for adjuvant treatment of GIST patients with imatinib mesylate.

## PATIENTS AND METHODS

Methodology for GIST data collection and analysis has been described in detail elsewhere (Nilsson *et al*, 2005). In essence, a retrospective (1983–2000) population-based study revealed that for GIST patients who initially undergo complete tumour removal by surgery (R0 resection), tumour recurrence correlates with the following prognostic factors: age, tumour size, time since surgery for GIST, Ki67 max% (immunohistochemical measure of proliferative index using monoclonal KIT antibody Ki-67) and deletion of exon 11 of the *KIT* gene.

Quality adjusted life years are the arithmetic product of life expectancy and a measure of the quality of the remaining life years, and place a weight on different health states. One year of perfect health is worth 1 QALY, and a year of less than perfect health life expectancy is worth less than 1 QALY. Death is considered to be equivalent to 0. Age- and sex-dependent QALY values are available in 5-year intervals (Kind, 1996): QALY values equal 1 per year for ages up to 50 years, and then successively decline with age to 0.76 and 0.67 QALY per year for 85-year-old men and women respectively. Similarly, QALY decreases are expected for GIST patients who suffer tumour recurrence. Additional treatment costs relating to tumour recurrence can be calculated separately from QALY. Albeit, here we make the assumption that the extra cost per day relating to tumour recurrence is a certain fixed proportion of the QALY generating cost for a GIST patient who has not suffered tumour recurrence at the same age. Consequently, the QALY function *q* is stepwise constant in 5-year intervals in our calculations, and the QALY value for an individual from age *a* to age *b* is defined as 
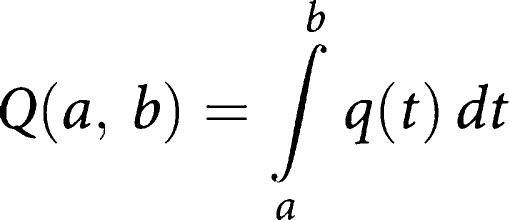
 If we take into account a possible QALY decrease following tumour recurrence at age *c* to a proportion *r*, and also include a discount of *p*%, the QALY is calculated as 
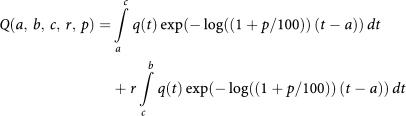
 For GIST patients who have not suffered tumour recurrence at age *b*, the corresponding QALY equals *Q(a,b,b,r,p)*, that is, *c=b.* The constant *r* is a merger that accounts for treatment side effects, reductions in health-related quality of life, and treatment costs following tumour recurrence at *c* (see Supplementary Information for details). Here we assume a 20% QALY decrease (*r*=0.80) following tumour recurrence at *c*.

Our main objective was to establish general methodology that permits more precise QALY calculations. Here we adapt the methodology to calculate the expected gain in QALYs for GIST patients following 1 year of adjuvant treatment with imatinib mesylate. To describe the instant rates of certain events, we define four hazard functions. The first, denoted *h*_r+d_, represents the combined event of detection of tumour recurrence, including local recurrence or metastases, or death before detection of tumour recurrence. This hazard function can be approximated by the sum of two separate hazard functions, *h*_r_, which represents detection of tumour recurrence, and *h*_d_, which represents death before detection of tumour recurrence. The *h*_r_ hazard function was estimated as a function of the prognostic factors for GIST listed above: current age, tumour size, time since surgery, Ki67 max% and the zero-or-one variable ‘*KIT* exon 11 deletion’. The *h*_d_ hazard function, representing death before detection of tumour recurrence, was estimated to be 8% above the death rate of the general population, and was put to 1.08 times the death rate of the general population. A fourth hazard function, denoted *h*_dar_, represents death after detection of tumour recurrence. Finally, the hazard function representing death among the general population was calculated from data available from Statistics Sweden (www.scb.se). All hazard functions are of the form exp(*β*_0_+*β*_1_*x*_1_+…+*β*_k_*x*_k_), where the *β’*s are coefficients and *x*_1_,…, *x*_*k*_ are the values of the variables, and were estimated by Poisson regression (Breslow and Day, 1987). A detailed derivation of the formula for QALY calculations using the hazard functions is available as Supplementary Information.

## RESULTS

Four hazard functions were considered to calculate the QALY gains resulting from adjuvant treatment of GIST patients with imantinib mesylate. Tables 1 and 2 and Supplementary Information Table I list the *β-*coefficients that define the hazard functions.

The variables Ki67 max%, tumour size and deletion of *KIT* exon 11 allow us to formulate a risk score for tumour recurrence, which equals the linear combination of the three variables with the weighted *β-*coefficients (Table 1). The estimated gradient of risk per 1 s.d. of the score is 4.21. This means that if two patients have risk scores that differ by 1 s.d., the patient with the higher score has 4.21 times higher risk of tumour recurrence than the patient with the lower score. Gradient of risk per 1 s.d. is a notion introduced to describe and compare the predictive power of a single variable or a score based on several variables. The gradient of risk, for example, of bone mineral density measured in the hip to predict hip fracture is 2.6 (Marshall *et al*, 1996). The risk of death without or before detection of tumour recurrence does not deviate significantly from the risk of death for the general population. The hazard ratio *vs* the general population is 1.08 (95%, confidence interval 0.89–1.36), whereas the risk of death after detection of tumour recurrence is substantially higher (Table 2).

**Figure 1:**
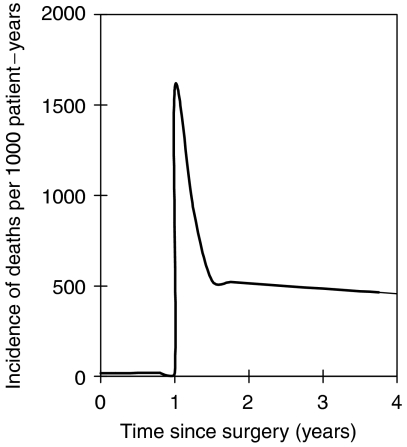
Illustration of the death hazard function and its dependence on tumour recurrence for GIST patients. In this example, a 65-year-old male undergoes complete tumour removal by surgery (R0 resection). Tumour recurrence is detected 1 year later. The probability of death, which is roughly proportional to the area under the curve, increases dramatically following tumour recurrence.

The death hazard function depicted in Figure 1 is an example that has been calculated from Tables 1 and 2, and the death hazard of the general population (Supplementary Information Table I). It shows how the risk of death changes for a 65-year-old man who undergoes surgery for GIST, and is diagnosed with tumour recurrence 1 year after the surgery. According to the above, his risk of death is very close to the risk of death for the general population before detection of tumour recurrence. In contrast, the risk of death increases dramatically following tumour recurrence.

Table 3 illustrates the expected QALY gains, adjusted for treatment costs following recurrence, resulting from administration of imatinib mesylate for four different GIST patients. The calculations reveal that treatment with imatinib mesylate results in considerably higher expected QALY gains for patients A and B than for patients C and D. The QALY gain exceeds 1 for patients A and B, while it is a moderate 0.25 and 0.20 for patients C and D (Table 3, yellow row). The calculations also reveal that larger QALY gains are accompanied by greatly reduced probability of death (Table 3, orange rows). The probability of death is reduced from 47.6 to 10% for patient B, who exhibits the highest calculated QALY gain (2.16), whereas the probability of death is reduced from 8.1 to 3.9% for patient D, who has a lower calculated QALY gain (0.20).

Expected QALY gains between 0.5 and 1.0 constitute typical limits for health care interventions in many Western countries, when the treatment cost to achieve the gain is in the interval US$ 40 000–80 000. We calculated the expected QALY gain for the GIST patients comprising the material in 0.5 year increments starting at the time of surgery. Only patients who were alive, and without detected tumour recurrence at the starting point, contributed to the calculation. The number of GIST patients with QALY gain limits above 1.00, 0.67 and 0.50 are listed in Table 4.

Only patients who were alive, and without detected tumour recurrence within the specified period after surgery, are available for adjuvant treatment. Assuming current health care legislation accepts treatment costs up to US$ 60 000 per 1 QALY gained, and that the actual cost for the treatment is 40 000, results in a QALY gain limit of 0.67 (40 000 out of 60 000). Table 4 shows that 11 patients are above a QALY gain limit of 0.67 two years after surgery. The total treatment cost then amounts to US$ 440 000 (11 × US$ 40 000). If the pharmaceutical company providing the drug would accept a lower price of US$ 30 000, the QALY gain limit decreases to 0.50 (30 000 out of 60 000). Table 4 shows that 17 patients are above a QALY gain limit of 0.50 two years after surgery. The total treatment cost now amounts to US$ 510 000 (17 × US$ 30 000). The lower drug price thus generates a 16% gross profit increase for the pharmaceutical company, while at the same time drug availability to patients is improved by 55%.

## DISCUSSION

Here we introduce new general methodology, that employs continuous hazard functions for analysis of survival data. To estimate the continuous hazard functions, we have used Poisson regression. This methodology means that short periods of time are considered and that the person years within a period are calculated, as well as the number of events. Each person can be treated separately, and the number of events will typically be 0 or 1 for each short period. A maximum likelihood function could be found and the parameters estimated by the same means (solving a nonlinear system of equations) as parameters in a Cox regression, or in a logistic regression. There are no restrictions on the number of parameters, so in principle any continuous function can be approximated. However, in practice the number of parameters has to be quite small because of the limited size of available materials for most cases.

The robustness of hazard function estimations, and the calculated expected QALY gains, can be determined by simulation. The hazard functions presented here permit simulation of events, so that new estimations can be determined, and expected QALY gains can be calculated repeatedly. We recommend simulations to study the robustness of the model for each case. Such a sensitivity analysis is more important for patients who are close to the cost-benefit border.

Prospective QALY gains were calculated for 10-year periods following adjuvant treatment of GIST with imatinib mesylate. Available data to estimate the risk of death for GIST patients following imatinib mesylate administration are limited. Clinical practice nonetheless indicates that it is very close to normal. Notably, none of the GIST patients who have undergone R0 tumour resection at our department have been diagnosed with tumour recurrence following 1 year of treatment with imatinib mesylate. The hazard ratio *vs* the general population is 1.08 (95%, confidence interval 0.89–1.36). Accordingly, we substituted the death hazard for GIST patients following treatment with imatinib mesylate with the death hazard function of the general population times 1.08 in our calculations. Moreover, no further reductions in overall quality of life have been reported for GIST patients without detected tumour recurrence. The expected QALY gain resulting from imatinib mesylate administration is the difference between the calculated QALY when imatinib mesylate is administered and the expected QALY without administration of imatinib mesylate. For the time being, we approximate the calculated QALY when imatinib mesylate is administered with that of the general population.

The methodology presented here is applicable for newly diagnosed GIST patients as well as patients who underwent surgery for GIST before imatinib mesylate was available. The pool of GIST patients, for whom treatment is currently considered, is thus made up of patients with newly detected GISTs as well as patients who underwent surgery up to several years ago. Such heterogeneity in the clinical situation is nevertheless typical for all types of conditions for which more efficient treatment options are expected to become available. A new generation of more selective cancer drugs is beginning to reach the market, and the methodology can easily be adapted to calculate the expected QALY gain for each of these drugs.

At a fixed drug price the methodology can be used to decide who should receive treatment, and who should not, depending on health care legislation. Alternatively, the methodology can be applied to negotiate optimal drug pricing and to investigate health care intervention thresholds. Either way the methodology may help reduce uncertainty in health care resource allocation, and optimally renders all win situations that improve drug availability to patients, make the most of public expenditure on drugs and increase pharmaceutical company gross profits.

## Figures and Tables

**Table 1:** *β*-Coefficients for the hazard function *h*_r_, representing detection of tumour recurrence

**Variable**	***β***	**s.e.**	***P*-value**
Constant	−6.2060	1.1495	—
Age	0.0153	0.0152	0.3139
Time since surgery	−0.1426	0.0828	0.0851
Ki67 max%	0.1161	0.0148	0.0000
Tumour size	0.1216	0.0194	0.0000
If *KIT* exon 11 deleted=1, if not=0	0.9767	0.3313	0.0032

GIST=Gastrointestinal stromal tumours. A total of 220 GIST patients who initially underwent complete tumour removal by surgery (R0 resection) were analysed to calculate the *β*-coefficients defining *h*_r_. A total of 37 tumour recurrences were detected in 1305.8 patient years. Standard errors (s.e.) and *P*-values are listed for each *β*-coefficient.

**Table 2:** *β*-Coefficients for the hazard function *h*_dar_, representing death after detection of tumour recurrence

**Variable**	***β***	**s.e.**	***P*-value**
Constant	−3.21524	1.55063	—
Current age	0.05582	0.02066	0.0069
Sex, man=0, woman=1	−0.94956	0.40851	0.0201
Min (time since recurrence, 0.5)	−2.26048	0.94729	0.0170
Max (time since recurrence, −0.5, 0)	−0.11525	0.02982	0.0001

GIST=Gastrointestinal stromal tumours. The 37 GIST patients who suffered tumour recurrence after R0 resection (see Table 1 legend) were analysed to calculate the *β*-coefficients defining *h*_dar_. Among these 37 GIST patients, a total of 33 deaths occurred in 150.7 patient years. Standard errors (s.e.) and *P*-values are listed for each *β*-coefficient.

**Table 3:**
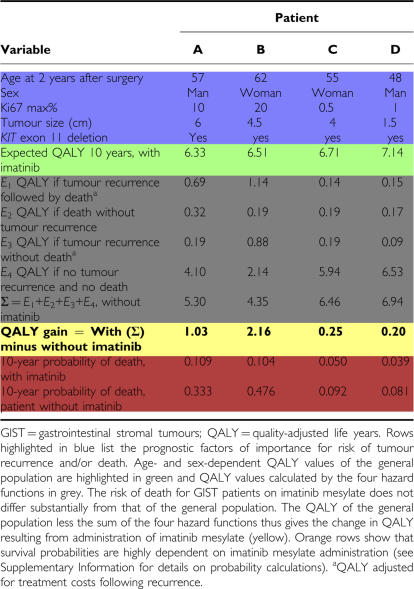
Ten-year prospective QALY gain calculations for four GIST patients that underwent R0 tumour resection 2 years ago

**Table 4:** Calculated number of patients who gain more than 1.0, 0.67 and 0.50 QALY if treatment with imatinib mesylate begins immediately after surgery (time since surgery 0.0 years) and if treatment with imatinib mesylate begins at later times following surgery (Time since surgery 0.5 years and upwards)

		**Number of patients above the limit**		
		**QALY gain limit**		
**Time since surgery (years)**	**Number of patients alive without recurrence**	**1.00**	**0.67**	**0.50**
0.0	220	31	39	45
0.5	204	24	31	38
1.0	187	14	20	28
1.5	178	10	14	20
2.0	171	8	11	17
2.5	161	2	6	10
3.0	152	2	5	8
4.0	149	2	3	5
5.0	143	2	3	5
6.0	139	1	1	3

QALY=quality-adjusted life year.
