# Supplementary material for: Reducing uncertainty in health-care resource allocation
Source: Br J Cancer. 2007 May 22;96(12):1834–8. doi: 10.1038/sj.bjc.6603795 (PMC2359979; doi:10.1038/sj.bjc.6603795)
Supplement: Supplementary Information [file 6603795x1.doc]

**Supplementary material**

*Derivation of a formula to calculate the expected gain in QALYs for treatment of GIST patients with imatinib mesylate*

If treatment of a GIST patient with imatinib mesylate is considered at a certain point in time, let *age_0_* represent the age of the patient at that point in time, and let *T* denote the time that has passed since the patient was diagnosed with GIST. Within a ten year time frame from *T*, the expected QALY without treatment with imatinib mesylate is the sum of four terms, *E*_1_*,…, E*_4_. The first term, *E*_1_*,* corresponds to detection of tumour recurrence at time *T+t* followed by death at *T+t+v*, where *t* and *v* runs through all possible combinations within the given time frame. The second term, *E*_2_*,* corresponds to death before detection of tumour recurrence at time *v*. The third term, *E*_3_*,* corresponds to detection of tumour recurrence at time *t* and survival to *T+10*. The fourth term, *E*_4_*,* corresponds to survival from *T* to *T+10* without detection of tumour recurrence.

As described in Materials and Methods we consider four hazard functions to calculate the expected gain in QALYs for treatment of GIST patients with imatinib mesylate. In short, the *h*_r+d_ hazard function represents the combined event of detection of tumour recurrence and death before detection of tumour recurrence. This hazard function can be approximated by the sum of two separate hazard functions, *h*_r_, which represents detection of tumour recurrence, and *h*_d_, which represents death before detection of tumour recurrence. Finally, the *h*_dar_ function represents death after detection of tumour recurrence, which in the adaptation for GIST specific QALY calculations turns out to depend on ‘current age’ and ‘time since detection of tumour recurrence’, but not on ‘time since surgery’. Therefore we only use ‘time since detection of tumour recurrence’ as a time variable in the argument below. ‘Current age’ and other variables, except the important time variables, have been omitted in the following notations.

$$\begin{matrix} E_{1}=\int_{0}^{10} \int_{t}^{10} Q(age_{0},age_{0}+t+v,age_{0}+t,r,p)\cdot\exp(-\int_{0}^{t} h_{r+d}(T+x)dx)\cdot h_{r}(T+t) \\ \cdot\exp(-\int_{t}^{v} h_{dar}(z)dz)\cdot h_{dar}(v)dvdt \end{matrix}$$

$$E_{2}=\int_{0}^{10} Q(age_{0},age_{0}+v,age_{0}+v,r,p)\cdot\exp(-\int_{0}^{v} h_{r+d}(T+z)dz)\cdot h_{d}(T+v)dv$$

$$\begin{matrix} E_{3}=\int_{0}^{10} \int_{t}^{10} Q(age_{0},age_{0}+t+v,age_{0}+t,r,p)\cdot\exp(-\int_{0}^{t} h_{r+d}(T+x)dx)\cdot h_{r}(T+t) \\ \cdot\exp(-\int_{t}^{10} h_{dar}(z)dz)dvdt \end{matrix}$$

$$E_{4}=Q(age_{0},age_{0}+10,age_{0}+10,r,p)\cdot\exp(-\int_{0}^{10} h_{r+d}(T+x)dx)$$

The terms *E*_1_ and *E*_3_ take into account detection of tumour recurrence at *T+t*, whereas the terms *E*_2_ and *E*_4_ are independent of this event.

If the QALY function *Q* is substituted for a function *Q(a,b,c,r,p*) = *b – a* , the sum of the four terms, *E*_1_*,…, E*_4_, gives the expected time of survival within the ten year time frame. If we put *Q*1, the sum equals the probability of survival.

The formula giving *E*_1_ can be briefly explained as follows: The factor$\exp(-\int_{0}^{t} h_{r+d}(T+x)dx)\cdot h_{r}(T+t)\cdot\Delta$, where Δ represents the length of a short interval, gives the probability of neither death, nor detection of tumour recurrence, from time *T* to time *T+t*, followed by detection of tumour recurrence in the short interval following *T+t*. Similarly, for *E*_2_, the expression $\exp(-\int_{t}^{v} h_{dar}(z)dz)\cdot h_{dar}(v)$ multiplied by the length of a short interval gives the probability of surviving the period from time *t* to time *v* after detection of tumour recurrence, followed by death in the short interval following *v*. The combined events of surviving without detection of tumour recurrence until time *t*, followed by detection of tumour recurrence at time *t* and death at time *v*, are disjoint events if *t* and *v* are different. So the probabilities can be added to give the probability of the union of the events when *t* and *v* run through all possible combinations. The sum tends to the integral when the short periods tend to zero. Similar explanations are applicable to *E*_2_, *E*_3_ and *E*_4_. Here we have used the approximation

*h*_r+d_ *= h*_r_ *+ h*_d_

and we have assumed that *h*_d_, the death hazard before detection of tumour recurrence, is equal to the death hazard of the general population. The hazard functions are of the form exp(*β*_0_ + *β*_1_·*x*_1_ + … + *β*_k_·*x*_k_), where the betas are coefficients and *x*_1_,…, *x*_k_ are the values of the variables.

*Death hazard of the general population*

# Supplementary material Table I. Age and sex specific risk of death among the general population*.* Beta coefficients were calculated from data available from Statistics Sweden (www.scb.se).

| **Variable** | **β men** | **β women** |
| --- | --- | --- |
| Constant | -7.6634 | -8.4138 |
| Calendar time since 1986 (years) | -0.0505 | -0.0351 |
| Min(age, 50) | 0.0404 | 0.0441 |
| Max(Min(age-50, 70-50),0) | 0.1114 | 0.1032 |
| Max(age-70, 0) | 0.0948 | 0.1157 |
| Age × calendar time since 1986 | 0.000479 | 0.000332 |
